# Effect of COVID-19 lockdown on health of women in Pakistan - Socioeconomic perspective

**DOI:** 10.12669/pjms.40.3.8312

**Published:** 2024

**Authors:** Amena Moazzam Baig, Musarrat Riaz, Amani Moazzam, Iram Zehra Bokharey

**Affiliations:** 1 Amena Moazzam Baig, FRCP Assistant Professor of Endocrine, Department of Endocrine and Metabolism, Services Institute of Medical Sciences, Lahore, Pakistan; 2 Musarrat Riaz, FCPS Associate Professor, Department of Medicine, Consultant Endocrinologist, Baqai Institute of Diabetology and Endocrinology, Baqai Medical University, Karachi, Pakistan; 3 Amani Moazzam, Ph.D. Assistant Professor, Institute of Administrative Sciences, University of the Punjab, Lahore, Pakistan; 4 Iram Zehra Bokharey, Ph.D. Consultant Clinical Psychologist, Tanwir Ahmad Medical Complex, M.M. Alam Road, Gulberg, Lahore, Pakistan

**Keywords:** Women health, Pandemics, COVID-19, Lockdown

## Abstract

**Objectives:**

This study investigates the dimensions of women’s experiences during the COVID-19 lockdown in Pakistan, considering their historical vulnerability to natural disasters.

**Methods:**

A cross-sectional study was conducted in Pakistan from May to September 2020 at Services institute of medical Sciences, Lahore. An online survey collected socio-demographic data, household responsibilities, and access to medical services using a self-designed questionnaire. Women aged 18 years and above (n=1307) were included through convenience sampling. Data analysis utilized SPSS 20.0.

**Results:**

Of the surveyed women, 10 (14.9%) experienced improved health outcomes, while 39 (58.27%) faced poor health outcomes. Proper access to medical services was reported by 29 (43.3%) participants, while 38 (57.1%) had no access. Two women (3.0%) conceived during the lockdown. 45 (67.2%) women lived in nuclear families, and 21 (31.3%) in joint family systems. Additionally, 46 (68.7%) women were significantly burdened with household chores, while 21 (31.3%) had a normal routine. Among COVID-19-positive respondents, 70% reported weight gain from increased screen time and sedentary lifestyle. Difficulties in managing children’s online classes were reported by 34.6% of participants. Moreover, 84% had a monthly income below one hundred thousand PKR. Among women aged 23-28 years, 30.9% had adverse effect on their husband’s income, and 4.7% experienced unemployment. Unfortunately, 16% of respondents lost a relative due to COVID-19. Even with access to health facilities.

**Conclusion:**

The COVID-19 lockdown in Pakistan led to adverse socioeconomic and health outcomes for women. These findings highlight the measures needed to address women’s challenges amid pandemic impact.

## INTRODUCTION

Amidst Covid, Pakistan implemented a nationwide lockdown lasting less than two months, beginning on March 16, 2020. Although these lockdown measures played a role in flattening the infection curve, they also had potential drawbacks. These included mental stress associated with social isolation and distancing, fears of falling ill, and concerns about job loss.^1^,^2^ Several risk factors have been identified that contribute to the increased severity of the disease and higher mortality rates.^3^ While both men and women faced an equal risk of contracting the virus, women had to bear the socio-economic consequences of the pandemic.^4^

Women, who make up 51% of the population in Pakistan, often find themselves marginalized and neglected. Numerous studies conducted worldwide have highlighted the mounting psychosocial pressures experienced by women during the pandemic.^5^ In times of epidemics, resources are often redirected from routine healthcare services towards managing the outbreak. As a result, women face limited access to crucial sexual and reproductive health services.^6^,^7^ As per Pakistan Bureau of Statistics for 2018-2019, only 22.9% of women aged 10 years and above in the country are employed, encompassing both low-skilled and skilled labor. (REF Pakistan Bureau of Statistics) This employment group was particularly vulnerable during the lockdown period.^8^

These women faced significant financial dependence on their husbands or siblings, which added to their mental and financial stress. Moreover, in another study done in Pakistan, negative effects of vaccination in the recipients were observed in the form of malaise, headaches and fever and were more common among patients presenting with comorbidities.

While numerous global studies have examined the impact of the current pandemic on women, there is a paucity of research in Pakistan Therefore, the objective of this study was to analyze the socio-economic factors in relation to women’s health during the lockdown in Pakistan.

## METHODS

This cross-sectional survey was conducted from May to September 2020 at Services Institute of Medical Sciences, Lahore. Data was collected from different cities of Pakistan through online questionnaire.

### Ethical Approval

IRB approval to conduct the study was taken from Services Institute of Medical Sciences Research cell, Ref No. IRB/2020/679/SIMS.

The study population comprised of women aged 18 years and above, both healthy and visiting the outdoor department of the hospital in addition to women who were contacted online via convenient sampling. The sample size was calculated by using Raosoft sample size calculator^9^ (http://www.raosoft.com/samplesize.html), based on 3% margin of error, 97% confidence interval, and 50% response distribution. The calculated sample size was 1307. The sample was collected using Convenience sampling design and a final sample of 1322 was collected.

After ethical approval, the data was collected using a self constructed online questionnaire^10^ at Diabetic center of Services institute of medical Sciences and Baqai Institute of Diabetology & Endocrinology, Karachi. Those participants who fulfilled the inclusion criteria were questioned. The participation in the study was exclusively voluntary after an informed consent. The questionnaire was shared by the researchers online with colleagues and participants of the study. A pilot study was done in which two interns were trained and supervised for data collection, from either not educated/without access to mobile apps women. Questionnaire was distributed among 2500 proposed participants with a response rate of 52%.

A self- constructed online questionnaire comprised of 50 questions which included socioeconomic details, gynecological history, weight changes, daily life chores and dietary habits. Perception regarding their health and preventive measures taken were also discussed. Parameters like number of children, the sleep patterns, level of exertion and screen time and how they affected their health were evaluated.

### Statistical Analysis

Data were stored and analyzed using IBM-SPSS version 23.0. Percentages were reported on age group, Education, Location, Family system, Employment, marital status and income. Outcome on covid-19 test were reported, these findings were associated with baseline characteristics, life style factors, general health, daily life activities, food items, meal intake, sleeping habits using Pearson Chi Square test. P-values less than 0.05 were considered statistically significant.

## RESULTS

The pooled cross-sectional sample of 1307 women were used. Among which 67(5.1%) of women reported to be COVID POSITIVE, 1174(88.9%) were COVID NEGATIVE and 80 (6.1%) didn’t undergo any investigation. The ages of women included in this study was from 18 years to 65 years. In some of the women weight fluctuations were observed during lockdown, 742(56.17%) of women showed weight gain while 163(12.34%) lost weight and 416 (31.49%) showed no weight changes. Majority of the women were responsible for looking after their loved ones during lockdown. Financially, people had to endure economic crisis and the effects of COVID 19 on average income were not good which results in increased mental stress. In our survey, total of 408 (30.9%) were the subjects whose husband’s income were affected badly and 157(11.9%) of subjects didn’t lose their jobs and were doing their job online but unfortunately 62(4.7%) were unemployed and 31 (2.3%) had to suspend their work in lockdown which affected their income adversely, (Table-I).

**Table-I:** Demographics of women during Covid-19 Lockdown.

Variable	Description	Frequency (%age)
Age bracket	18-22	126 (9.5%)
	23-28	282 (21.3%)
	29-35	229 (17.3)
	35-43	226 (17.1%)
	43-50	236 (17.9%)
	above	222 (16.8%)
Weight change	Above	742 (56.17%)
	Below	163 (12.34%)
	Stable	416 (31.49%)
Educational level	Uneducated	358 (27.1%)
	Educated	963 (72.9%)
Taking care of	Children	421 (31.9%)
	Parents	172 (13.0%)
	Both	517 (39.1)
	Not applicable	197 (14.9%)
	One with special care	14 (1.1%)
Income status	Less than Rs. 20,000	1155 (87.4%)
	Between Rs. 20,000 - 100,000	127 (9.6%)
	Between Rs. 100,000 - 250,000	39 (3.0%)
Marital status	Single	280 (21.2%)
	Married	972 (73.6%)
	Widow	45 (3.4%)
	Divorced	24 (1.8%)
Employment status	Unemployed	62 (4.7%)
	Housewife	393 (29.8%)
	Work from home	157 (11.9%)
	Suspended the work	31 (2.3%)
	Work as usual	120 (9.1%)
	Student	97 (7.3%)
Effect of COVID-19 on average income	Husband income is affected during lockdown	408 (30.9%)
	Siblings’ income is affected during lockdown	53 (4.0%)
COVID-19 infection	Yes	67 (5.1%)
	No	1174 (88.9%)
	Not Done	80 (6.1%)

Lifestyle in lockdown was greatly influenced secondary to various factors and women were observed to be predominantly affected due to their family structure, as 45 (67.2) % were living alone with their children and husband, 21(31.3%) were living in joint family and consequently leads to increased burden of home chores.However, 46(68.7%) subject showed increased intensity of household work whereas, 21(31.3%) showed decreased intensity. Lockdown had augmented sedentary lifestyle and lead to impaired physical and mental health. Sleep-time was observed to be increased with limited physical activities. Mental health was getting affected as 194 subjects lost their loved ones or seen them suffering secondary to infection, 183(35%) women had difficulty in managing their children’s studies and online schedule, (Table-II).

**Table-II:** Life style of women during COVID-19 Lockdown.

Were you tested positive COVID-19?					

Variables		Yes	No	Not Done	P-value

		N (%)	N (%)	N (%)	
Family System	Nuclear family	45 (67.2%)	625 (53.2%)	49 (61.3%)	<0.01
	Joint family	21 (31.3%)	541 (46.1%)	25 (31.3%)	
Responsible for Care at home	Parents	23 (34.3%)	129 (11.0%)	20 (25.0%)	<0.01
	Children	30 (44.8%)	372 (31.7%)	19 (23.8%)	
	Both	08 (11.9%)	499 (42.5%)	10 (12.5%)	
	Children with special care	01 (1.5%)	11 (0.9%)	02 (2.5%)	
	Not applicable	05 (7.5%)	163 (13.9%)	29 (36.3%)	
No. of dependent children	Age less than 16 years	23 (34.3%)	335 (28.5%)	14 (17.5%)	<0.01
	Age more than 16 years	16 (23.9%)	230 (19.6%)	13 (16.3%)	<0.01
Total duration of household work per day	1-3 hours	34 (66.7%)	777 (73.2%)	33 (50.0%)	<0.01
	3-6 hours	16 (31.4%)	231 (21.8%)	18 (27.3%)	
	More than 6 hours	01 (2.0%)	53 (5.0%)	15 (22.7%)	
Intensity of household work increased during lockdown?	Yes	46 (68.7%)	773 (65.8%)	40 (50.0%)	<0.01
	No	21 (31.3%)	401 (34.2%)	40 (50.0%)	
Sleep time during lockdown.	Increased	15 (22.4%)	212 (18.1%)	30 (37.5%)	<0.01
	Decreases	19 (28.4%)	199 (17.0%)	15 (18.8%)	
	Same	33 (49.3%)	763 (65.0%)	35 (43.8%)	
Change in no.of meals per day	increased	29 (43.3%)	422 (35.9%)	40 (50.0%)	0.02
	decreased	38 (56.7%)	752 (64.1%)	40 (50.0%)	
Difficulty coping with online schoolwork of your children?	yes	14 (20.9)	169 (14.4)	09 (11.3)	<0.01
	No	14 (20.9)	525 (44.7)	7 (8.8)	
	not applicable	39 (58.2)	480 (40.9)	64 (80.0)	
Close friend or family member tested positive for COVID-19 and died?	yes	40 (59.7)	154 (13.1)	12 (15.0)	<0.01
	no	26 (38.8)	989 (84.2)	55 (68.8)	
	maybe	01 (1.5)	31 (2.6)	13 (16.3)	

*p<0.05 was considered statistically significant using Pearson Chi Square test.

Overall, lockdown has shown a declining curve on general health of women as 559 (42.7%) of women were deteriorated physically and emotionally. Although, 687 of subjects had access to medical care. In terms of pregnancy, 141(%) of women conceived during lockdown (Table-III). Pearson Chi Square test did give a significant association of all these baseline characteristics with COVID-19 positivity, p<0.05. Table-III reports the general health of women among COVID-19 positive females 26.9% had easy access to contraceptive devices during lockdown, 91% had access to personal hygiene care devices.

**Table-III:** General health of women during COVID-19 LOCKDOWN.

Variables		Were you tested positive COVID-19?			P-value

		Yes	No	Not Done	

		N (%)	N (%)	N (%)	
Respect COVID-19 Restrictions?	Yes	60 (89.6%)	1118 (95.2%)	67 (83.8%)	<0.01
	No	07 (10.4%)	56 (4.8%)	13 (16.3%)	
General Health	Improved	10 (14.9%)	151 (12.9%)	12 (15.0%)	<0.01
	Deteriorated	39 (58.2%)	553 (47.1%)	25 (31.3%)	
	Same	18 (26.9%)	470 (40.0%)	43 (53.8%)	
Access to medical care during lockdown?	Yes	29 (43.3%)	658 (56.0%)	28 (35.0%)	<0.01
	No	38 (56.7%)	516 (44.0%)	52 (65.0%)	
Pregnancy during lockdown.	Yes	02 (3.0%)	139 (11.8%)	03 (3.8)	<0.01
	No	65 (97.0%)	1035 (88.2%)	77 (96.3%)	

## DISCUSSION

The study aimed to assess the consequences of lockdown on women’s health who tested COVID positive. It was observed that 89.6% of the respondents respected the lockdown restrictions. The study is unique in the sense that it was focused on women, one of the marginalized and vulnerable segments of the society, more prone to COVID as they were likely to be engaged as caregivers to patients infected at home and often without adequate protection.^11^,^12^The lockdown along with closure of schools led to shortage in available job opportunities causing high percentage of underpaid /lower waged employee.^13^

Many relief schemes and support programs were run by government to support individuals who were unemployed. The financial constraints were particularly prominent among the younger age group (between 23-28 years) belonging to the lower-middle socioeconomic class. This finding aligns with a recently published study that reported unemployment rates among Pakistani youth, although relatively lower compared to other South Asian countries.^14^ Data collected on the physical activity, screen time, and diet of women during the lockdown period shows majority of the respondents were less active and engaged in mild physical exertion.^15^ The reported maximum duration of daily household chores was three hours. Additionally, the participants reported spending at least six hours per day on screens, including TV, laptops, mobile devices, and online classes. Body image and weight play significant roles in women’s health. Health surveys conducted in South Asian countries and other nations have consistently shown a trend of weight gain among the female population, which aligns with the findings of our study (70.1% weight gain).^16^,^17^ Access to junk food, limited physical activity, and an increased number of meals consumed by mothers during the lockdown period may have contributed to this weight gain.^18^-^21^ Despite the implementation of a lockdown, emergency services in hospitals and pharmacies remained easily accessible to the public. When directly questioned, 50% of the respondents acknowledged the ease of accessing physicians and gynecologists; however, they reported experiencing poor health conditions.^22^-^24^ Despite of having access to health services, some of the people stayed at home and did self-medication, saturation at hospitals and covid units delayed the treatment.

The limited government-sponsored COVID testing resulted in a low positive test rate of 5%, but approximately 20% of respondents reported that their family members had been infected, as they exhibited clinical symptoms indicative of COVID and were unable to undergo testing due to saturation, unaffordability or unavailability of biochemical tests, contributing to the underreporting of cases. The majority of the population adopted preventive measures during the lockdown, yet many reported a deterioration in their health during this time.

### Limitations

A cross sectional survey was performed instead of a case control study design due to unavailability of data before the lockdown for comparison. Despite easy access, study couldn’t assess if respondents had true autonomy in using contraceptives and personal hygiene devices. History regarding domestic violence was also not clearly evaluated due to cultural and social constraints.

## CONCLUSION

COVID-19 lockdown during first wave had substantial financial constraints on the lives of women. Their added responsibility at home led them to perceive that their health had deteriorated during lockdown. This study helps to identify potential areas that women need to focus to maintain their lifestyles during the continued pandemic and lockdown situation.

### Authors contribution:

**MA:** Concept and design, interpretation of data, edited and approved the final manuscript.

**RM:** Data collection, Manuscript writing, final editing.

**BA and BI:** Data collection, Manuscript writing, approved the final manuscript.

**MA and RM**: are responsible and accountable for the accuracy or integrity of the work.
